# ggClusterNet: An R package for microbiome network analysis and modularity‐based multiple network layouts

**DOI:** 10.1002/imt2.32

**Published:** 2022-06-13

**Authors:** Tao Wen, Penghao Xie, Shengdie Yang, Guoqing Niu, Xiaoyu Liu, Zhexu Ding, Chao Xue, Yong‐Xin Liu, Qirong Shen, Jun Yuan

**Affiliations:** ^1^ Jiangsu Provincial Key Lab for Organic Solid Waste Utilization, Key Laboratory of Green Intelligent Fertilizer Innovation, Jiangsu Collaborative Innovation Center for Solid Organic Wastes, Educational Ministry Engineering Center of Resource‐Saving Fertilizers Nanjing Agricultural University Nanjing China; ^2^ Nanjing Meta Biotechnology Co., Ltd. Nanjing China; ^3^ State Key Laboratory of Plant Genomics, Institute of Genetics and Developmental Biology, The Innovative Academy of Seed Design Chinese Academy of Sciences Beijing China

**Keywords:** microbiome, network analysis, R package, visualization

## Abstract

The network analysis has attracted increasing attention and interest from ecological academics, thus it is of great necessity to develop more convenient and powerful tools. For that reason, we have developed an R package, named “ggClusterNet,” to complete and display the network analysis in an easier manner. In that package, ten network layout algorithms are designed to better display the modules of microbiome network (randomClusterG, PolygonClusterG, PolygonRrClusterG, ArtifCluster, randSNEClusterG, PolygonModsquareG, PolyRdmNotdCirG, model_Gephi.2, model_igraph, and model_maptree). For the convenience of the users, many functions related to microbial network analysis, such as corMicor(), net_properties(), node_properties(), ZiPiPlot(), random_Net_compate(), are integrated to complete the network mining. Furthermore, the pipeline function named network.2() and corBionetwork() are also added for the quick achievement of the network or bipartite network analysis as well as their in‐depth mining. The ggClusterNet is publicly available via GitHub (https://github.com/taowenmicro/ggClusterNet/) or Gitee (https://gitee.com/wentaomicro/ggClusterNet) for users' access. A complete description of the usages can be found on the manuscript's GitHub page (https://github.com/taowenmicro/ggClusterNet/wiki).

## Highlights

- ggClusterNet is an R package for microbial networks.
- Analysis functions could help the user to easily complete network analysis and interpretation.
- Ten network layout algorithms allow users more alternatives to plot the network and generate published‐ready figures.
- It is free to access on GitHub and Gitee.

## Graphical abstract

ggClusterNet is an R package for microbial networks analysis and interpretation. Ten network layout algorithms  allow users more alternatives to plot the network and generate published‐ready figures. It is free to access on GitHub and Gitee.

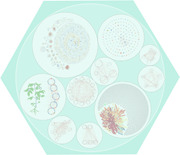

## INTRODUCTION

In the past two decades, the rapid development of high‐throughput sequencing technology has contributed to progress in microbial community studies as well as related bioinformatics tools [1]. Among the important and common analysis methods of microbiome, network analysis and network thinking [2] have been widely used by biologists, mathematicians, social scientists, and computational scientists to explore interactions between entities, be they individuals in a school [3], species in a food web [4], nodes on a computer network [5], proteins in metabolic pathways [6], and group comparisons in Venn network [7]. Network analysis has been used to explore the mathematical, statistical, or structural properties of a set of items (nodes) and the connections between them (edges [8]). It is also widely applied to the exploration of the co‐occurrence patterns between microbial taxa within complex communities. For example, Ma et al. highlighted the interconnection patterns across microbiomes in various environments, and emphasized the importance of the co‐occurrence feature of microbiomes with the network analysis [9]; Yuan et al. found that climate changes enhanced the complexity and stability of microbial networks [10].

Tools for network analysis of microbiome included web tool MENA [11] (MENAP), R packages (WGCNA [12], igraph [13], ggraph [14], SpiecEasi [15], interactive software (Cytoscape [16] and Gephi [17]), python packages (NetworkX [18] and SparCC [19]), and so forth. Many tools can be employed in the construction of the networks, for example, MENA was specifically designed for microbiome data and was easy to implement and robust against noise based on Random Matrix Theory (RMT) method; WGCNA was used to construct a scale‐free topology weighted gene network based on a soft thresholding power; SpiecEasi could combine data transformations developed for compositional data analysis with a graphical model inference framework and accompanied by a set of computational tools to generate operational taxonomic units (OTUs) count data from a set of diverse underlying network topologies. Some tools, such as Cytoscape, Gephi, and R packages (igraph, ggraph, etc.) integrated the function of network visualization. Cytoscape could not only provide a wide range of powerful visualization schemes but also allow users to develop new features with many plugins; Gephi could easily complete a quite aesthetic visualization of the network with few operation steps. Visualization tools of R packages (igraph, ggraph) could also display the network in the command line with less time, reproducibly. However, these tools were incapable to complete the job of network visualization in all aspects easily, such as providing multiple visualization layouts, rapid calculation of results, and repeating easily. For example, Cytoscape had tedious steps and was not easily reproducible; the layouts in igraph and ggraph were not aesthetically appealing enough.

**Figure 1:**
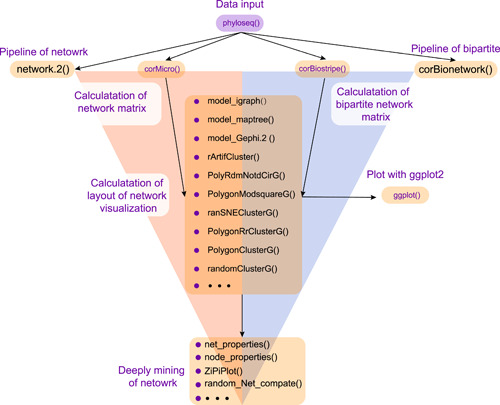
The workflow and tools exhibit the steps of network construction and analysis in ggClusterNet

Currently, more researchers have focused on the interactions among microbial network modules, to explore their functions. However, these tools suffered from large difficulties associated with too many connections within a variety of microbial species in the network and the lack of suitable visualization schemes and software. To address the rise in demands for network analysis and visualization, we have developed the R package ggClusterNet via R Language [20]. It provides a fast, automated, and easy‐to‐use pipeline for network analysis with multiple powerful visualizations (Figure 1). The ggClusterNet has the following characteristics: (1) The network analysis pipeline can complete quickly; (2) the network analysis pipeline can provide multiple exquisite visualization layouts; and (3) the network analysis pipeline can be completed with a few codes and is reproducible. It is expected that our tool could promote studies in the fields of microbiology and receive more collaborations with individuals and institutions for maintenance and development.

## METHODS

The ggClusterNet is a package and network pipeline, which was developed under R language environment. During the development of this package, the functions cor() and Pvalue() (in WGCNA packages), sparccboot() (in SpiecEasi packages) and corr.test() (in psych packages) were used for calculation of correlations with the references of layout algorithm in ggraph and sna. These functions (average.path.length(), edge.connectivity(), no.clusters(), centralization.closeness(), erdos.renyi.game(), etc.) in igraph were used for deep mining networks. All the scripts were deposited in GitHub https://github.com/taowenmicro/ggClusterNet/. This package can be installed by *devtools::install_github("taowenmicro/ggClusterNet")* command in R.

To facilitate the use of multiple visualization layouts and the pipeline of network analysis, we developed a freely available R package, named ggClusterNet by connecting network visualization and deep mining (Figure 1). Ten network visualization layout algorithms were encapsulated in ggClusterNet as functions: randomClusterG, PolygonClusterG, PolygonRrClusterG, ArtifCluster, randSNEClusterG, PolygonModsquareG, PolyRdmNotdCirG, model_Gephi.2, model_igraph, and model_maptree, respectively. These functions can be invoked individually. All these layout algorithms require correlation matrices as input, thus ggClusterNet provides the functions of corMicro() and corBigMicro(), which could compute multiple correlation matrices, including “spearman”, “pearson,” “kendall,” and “sparcc,” to satisfy the layouts needs.

To make the ggClusterNet available for users, we have made our R packages publicly available via GitHub (https://github.com/taowenmicro/ggClusterNet/) and Gitee (https://gitee.com/wentaomicro/ggClusterNet). More information, including a user guide, example script, and an extensive wiki, can be found on GitHub. A complete description of the data and usages can be found on the manuscript's GitHub page (https://github.com/taowenmicro/ggClusterNet/wiki).

## RESULTS

### Workflow in ggClusterNet

**Figure 2:**
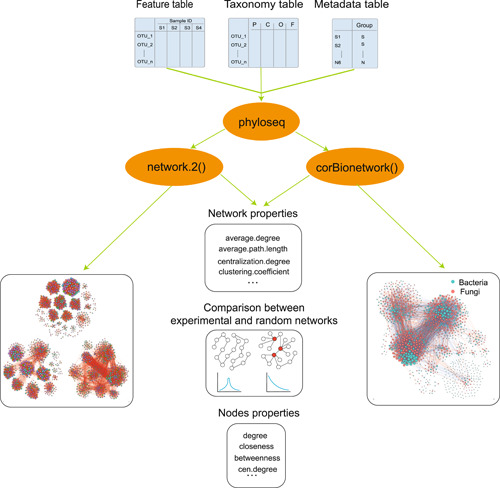
The workflow of the pipeline function names network.2() and corBionetwork() included input data type and output results

In the ggClusterNet, corMciro() (correlation matrices calculation for microbiome networks) or corBiostripe() [21] (correlation matrices calculation for bipartite networks) were used for calculating the correlation matrices (Figure 1). More than 10 layout algorithms were designed to calculate the layout of the visualization and plotted with ggplot2. The net_properties(), node_properties(), and ZiPiPlot() were integrated to calculate network properties, calculate node properties, and the role of a node according to modules, respectively. The random_Net_compate() was implanted to generate random networks following the null model and compared network properties within the experiment network (Figure 1). These functions were all included in the network.2() (pipeline for microbiome network) or corBionetwork() (pipeline for bipartite network) (Figure 2). Together, ggClusterNet can complete whole microbiome and bipartite network analysis from correlations calculation, network visualization, network properties calculation, and node properties and construction of the random networks and comparation.

### Network layout

**Figure 3:**
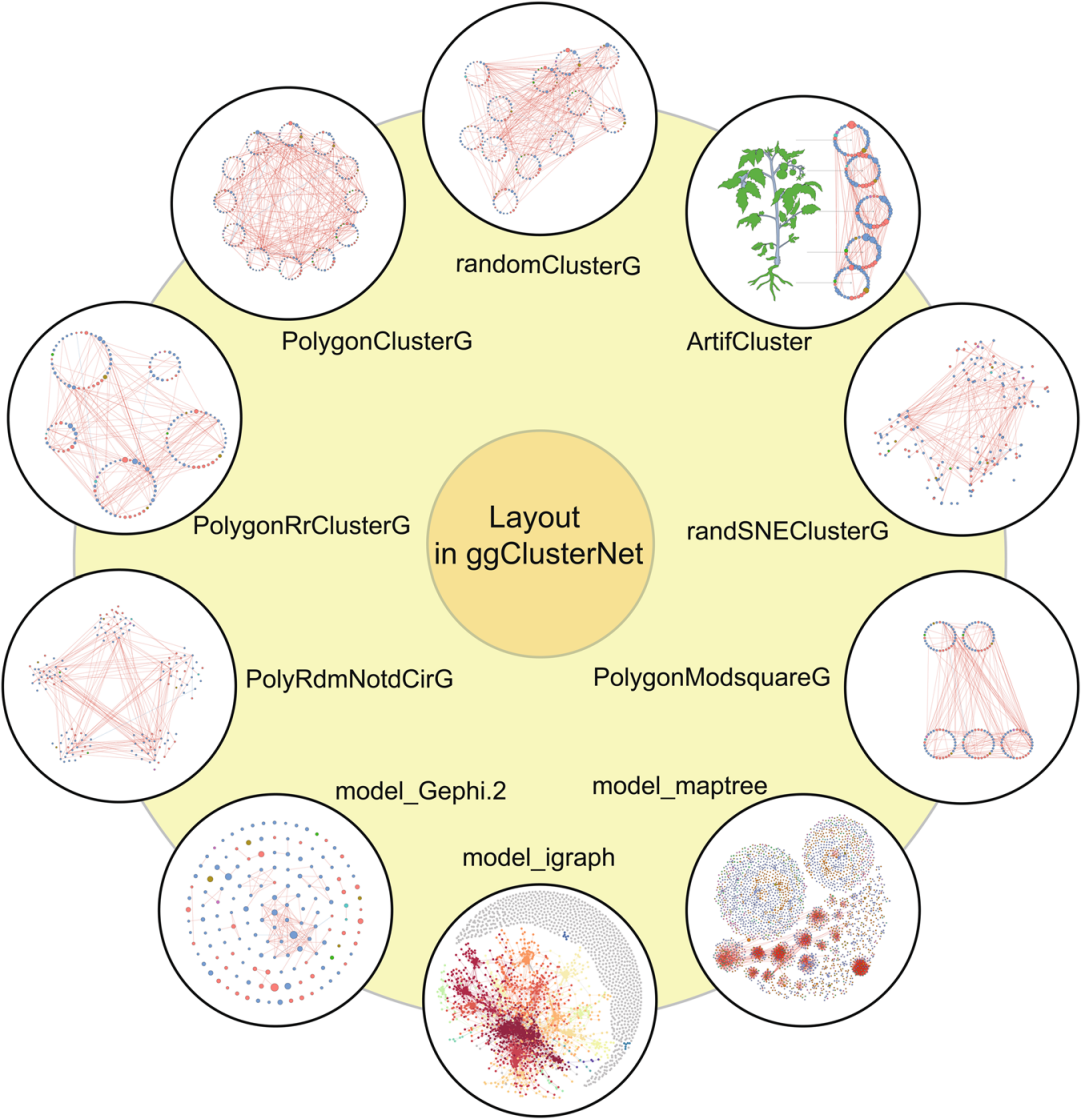
Ten layout algorithms of network visualization in ggClusterNet

For better visualizing the microbial networks and highlighting the modules, we developed 10 layouts algorithms for visualization (Figure 3). These functions of algorithms are presented below:

**randomClusterG**: Nodes of a module (group) were all arranged into one ring. Multiple modules were plotted as multiple same radii circles. Then, a function was designed, which could randomly arrange the rings in the plot panel.

**ArtifCluster**: Nodes of a module (group) were all arranged into one ring. Multiple modules were plotted as multiple same radii circles. Then, those circles were arranged manually by setting the coordinate values.

**randSNEClusterG**: Nodes of a module (group) were all arranged by multiple layouts in the sna package [22], respectively. Multiple modules were arranged randomly in the plot panel.

**PolygonModsquareG**: Nodes of a module (group) were all arranged into one ring. Multiple modules were plotted as multiple different radii circles (The higher number of nodes, the larger size of the radii). Then, those circles were arranged into one or more rows manually.

**model_maptree**: The modularity analysis was first conducted for the network and nodes grouped by network modularity and then were used for the calculation of coordinate. The relative position of the nodes was calculated based on the algorithm developed by Weixin Wang et al., which tried to find the densest packing of circles as they were added, one by one [23].

**model_igraph**: All nodes were placed on the plane using the force‐directed layout algorithm by Fruchterman and Reingold. Nodes with a high degree calculated with the R package igraph tended to be grouped, while nodes with a low degree were distributed within the surrounding network.

**model_Gephi.2**: All nodes were plotted as a circle and calculated the coordinates. Then, the coordinates value of each node was used to formulate clusters and re‐assigned to nodes.

**PolyRdmNotdCirG**: Nodes were randomly distributed in multiple different radii circles (the higher number of nodes, the larger size of the radii) according to module information. Then, those modules were arranged regularly to vertices of the polygon (The number of edges is equal to the number of modules) with the center of the origin of the coordinate axis.

**PolygonRrClusterG**: Nodes of a module (group) were arranged into one ring. Multiple modules were plotted as multiple different radii circles (the higher number of nodes, the larger size of the radii). Then, those circles were regularly arranged to vertices of the polygon with the center of the coordinate axis.

**PolygonClusterG**: Nodes of a module (group) were all arranged into one ring. Multiple modules were plotted as multiple same radii circles. Then, those circles were arranged regularly to vertices of the polygon (the number of edges is equal to the number of modules) with the center of the origin of the coordinate axis.

## DISCUSSION

Microbial ecology researchers have gradually favored network analysis [24], and thus, many powerful tools were developed, such as Cytoscape, Gephi, igraph, and so forth. In comparison with the previous network analysis tools, ggClusterNet showed significant advantages. Cytoscape and Gephi were popular for visualization due to their interactive graphical user interface and attractive visualization results. Cytoscape offered powerful functions for network analysis, though many parameters need adjustment for the module's visualization in the network. Gephi could display the modules in the network using default parameters, while less could be done of the deeply mining network. R packages (e.g., igraph, ggraph, sna) supplied many topological properties during network analysis, but their visualizations were less aesthetical than Gephi and Cytoscape generated. The ggClusterNet integrated the advantages of igraph, ggraph, and sna, and it provided the multiple layout algorithms, and its pipeline (network.2() and network()) can show the modules within networks and deep mining of the network.

The future work will continue to develop ggClusterNet. To enhance the function of microbiome network analysis, it will be added in the pipeline with the stability of network dynamics and deeply mining the module's function to maintain network stability. Then, shiny will be used to construct the user‐friendly interface, and it will be more convenient for more investigators to explore the networks in detail. In addition, further mining for bipartite network analysis and the development of more layout algorithms suitable for the bipartite network will be conducted in our future work.

## AUTHOR CONTRIBUTIONS

All authors contributed to the pipeline development and workflow of analyses. The initial idea and framework were conceived by Tao Wen, Yong‐Xin Liu, and Jun Yuan. The pipeline was built and maintained by Tao Wen, Peng‐Hao Xie, and Yong‐Xin Liu. During the pipeline development, Guo‐Qing Niu, Sheng‐Die Yang, Zhe‐Xu Ding, Xiao‐Yu Liu, and Chao Xue contributed to the pipeline test and usage for network analyses. This manuscript was written by Tao Wen and Peng‐Hao Xie, and revised by Yong‐Xin Liu, Jun Yuan, and Qi‐Rong Shen.

## CONFLICT OF INTEREST

The authors declare no conflict of interest.

## Data Availability

ggClusterNet is publicly accessible to all researchers on GitHub (https://github.com/taowenmicro/ggClusterNet/) or Gitee (https://gitee.com/wentaomicro/ggClusterNet), and users can download and install it in R (version > 3.6). Supplementary materials (figures, tables, scripts, graphical abstract, slides, videos, Chinese translated version, and update materials) may be found in the online DOI or iMeta Science http://www.imeta.science/.

## References

[1] Liu, Yong‐Xin , Yuan Qin , Tong Chen , Meiping Lu , Xubo Qian , Xiaoxuan Guo , and Yang Bai . 2021. “A Practical Guide to Amplicon and Metagenomic Analysis of Microbiome Data.” Protein Cell 12: 315–30. 10.1007/s13238-020-00724-8 32394199 PMC8106563

[2] Proulx, Stephen Robert , Daniel Promislow , and Patrick Phillips . 2005. “Network Thinking in Ecology and Evolution.” Trends in Ecology Evolution 20: 345–53. 10.1016/j.tree.2005.04.004 16701391

[3] Moody, James . 2001. “Race, School Integration, and Friendship Segregation in America.” American Journal of Sociology 107: 679–716. 10.1086/338954

[4] Krause, Ann , Kenneth Frank , Doran Mason , Robert Ulanowicz , and William Taylor . 2003. “Compartments Revealed in Food‐Web Structure.” Nature 426: 282–85. 10.1038/nature02115 14628050

[5] Pastor‐Satorras, Romualdo , and Alessandro Vespignani . 2001. “Epidemic Spreading in Scale‐Free Networks.” Physical Review Letters 86: 3200. 10.1103/PhysRevLett.86.3200 11290142

[6] Guimera, Roger , and Luis Nunes Amaral . 2005. “Functional Cartography of Complex Metabolic Networks.” Nature 433: 895–900. 10.1038/nature03288 15729348 PMC2175124

[7] Chen, Tong , Haiyan Zhang , Yu Liu , Yong‐Xin Liu , Luqi Huang . 2021. “EVenn: Easy to Create Repeatable and Editable Venn Diagrams and Venn Networks Online.” Journal of Genetics Genomics 48: 863‐66. 10.1016/j.jgg.2021.07.007 34452851

[8] Newman, Mark. 2003. “The Structure and Function of Complex Networks.” SIAM Review 45: 167–256. 10.1137/S003614450342480

[9] Ma, Bin , Yiling Wang , Shudi Ye , Shan Liu , Erinne Stirling , Jack A. Gilbert , Karoline Faust , et al. 2020. “Earth Microbial Co‐Occurrence Network Reveals Interconnection Pattern Across Microbiomes.” Microbiome 8: 82. 10.1186/s40168-020-00857-2 32498714 PMC7273686

[10] Yuan, Mengting Maggie , Xue Guo , Linwei Wu , Ya Zhang , Naijia Xiao , Daliang Ning , Zhou Shi , et al. 2021. “Climate Warming Enhances Microbial Network Complexity and Stability.” Nature Climate Change 11: 343–48. 10.1038/s41558-021-00989-9

[11] Deng, Ye , Yi‐Huei Jiang , Yunfeng Yang , Zhili He , Feng Luo , and Jizhong Zhou . 2012. “Molecular Ecological Network Analyses.” BMC Bioinformatics 13: 1–20. 10.1186/1471-2105-13-113 22646978 PMC3428680

[12] Langfelder, Peter , and Steve Horvath . 2008. “WGCNA: An R Package for Weighted Correlation Network Analysis.” BMC Bioinformatics 9: 1–13. 10.1186/1471-2105-9-559 19114008 PMC2631488

[13] Csardi, Gabor , and Tamas Nepusz . 2005. “The Igraph Software Package for Complex Network Research.” *InterJournal Complex Systems*, 1695.

[14] Si, Beibei , Yuxuan Liang , Jin Zhao , Yu Zhang , Xiaofei Liao , Hai Jin , Haikun Liu , and Lin Gu . 2020. “GGraph: An Efficient Structure‐Aware Approach for Iterative Graph Processing.” *IEEE Transactions on Big Data*. 10.1109/TBDATA.2020.3019641

[15] Kurtz, Zachary , Christian Lorenz Müller , Emily Miraldi , Dan. Littman , Martin Blaser , and Richard Bonneau . 2015. “Sparse and Compositionally Robust Inference of Microbial Ecological Networks.” *PLOS Computational Biology* 1–25. 10.1371/journal.pcbi.1004226 PMC4423992 25950956

[16] Shannon, Paul , Andrew Markiel , Owen Ozier , Nitin S. Baliga , Jonathan T. Wang , Daniel Ramage , Nada Amin , Benno Schwikowski , and Trey Ideker . 2003. “Cytoscape: A Software Environment for Integrated Models of Biomolecular Interaction Networks.” Genome Research 13: 2498–504. 10.1101/gr.1239303 14597658 PMC403769

[17] Bastian, Mathieu , Sebastien Heymann , and Mathieu Jacomy . 2009. “Gephi: An Open Source Software for Exploring and Manipulating Networks.” *Proceedings of the International AAAI Conference on Web and Social Media* 3: 361–62. https://ojs.aaai.org/index.php/ICWSM/article/view/13937

[18] Hagberg, Aric , Pieter Swart , and Daniel Chult . 2008. “Exploring Network Structure, Dynamics, and Function Using NetworkX.” https://www.osti.gov/servlets/purl/960616

[19] Maksymowych, Walter , Catherine Mallon , Sharon Morrow , Kamran Shojania , Wojciech Olszynski , Robert Wong , John Sampalis , and Barbara Conner‐Spady . 2009. “Development and Validation of the Spondyloarthritis Research Consortium of Canada (SPARCC) Enthesitis Index.” Annals of the Rheumatic Diseases 68: 948–53. 10.1136/ard.2007.084244 18524792

[20] Ihaka, Ross , and Robert Gentleman . 1996. “R: A Language for Data Analysis and Graphics.” Journal of Computational and Graphical Statistics 5: 299–314. 10.2307/1390807

[21] Feng, Kai , Xi Peng , Zheng Zhang , Songsong Gu , Qing He , Wenli Shen , Zhujun Wang , et al. 2022. “iNAP: An Integrated Network Analysis Pipeline for Microbiome Studies.” iMeta 1: e13. 10.1002/imt2.13 PMC10989900 38868563

[22] Butts, Carter Tribley . 2008. “Social Network Analysis with sna.” Journal of Statistical Software 24: 1–51. 10.18637/jss.v024.i06 18618019 PMC2447931

[23] Wang, Weixin , Hui Wang , Guozhong Dai , and Hongan Wang . 2006. “Visualization of Large Hierarchical Data by Circle Packing.” *Proceedings of the SIGCHI Conference on Human Factors in Computing Systems*, Association for Computing Machinery, Montréal, Québec, Canada, pp. 517–20. 10.1371/journal.pcbi.1004226

[24] Fuhrman, Jed A. 2009. “Microbial Community Structure and Its Functional Implications.” Nature 459: 193–99. 10.1038/nature08058 19444205

